# Microfluidic Gratings for X‐Ray Phase Contrast Imaging

**DOI:** 10.1002/advs.77561

**Published:** 2026-09-08

**Authors:** Alessandro Rossi, Antonio Ferraro, Francesco Coccimiglio, Tiziana Ritacco, Alberto Astolfo, Michele Giocondo, Vincenzo Formoso, Raffaele Giuseppe Agostino, Francesco Iacoviello, Ioannis Papakonstantinou, Alessandro Olivo

**Affiliations:** ^1^ Photonic Innovations Lab, Department of Electronic & Electrical Engineering University College London London United Kingdom; ^2^ Consiglio Nazionale delle Ricerche ‐ Istituto di Nanotecnologia CNR‐NANOTEC Rende Italy; ^3^ Department of Physics and STAR Research Infrastructure University of Calabria Rende Cosenza Italy; ^4^ Consiglio Nazionale delle Ricerche ‐ Istituto di Microelettronica e Microsistemi Rome Italy; ^5^ Department of Medical Physics and Biomedical Engineering University College London London United Kingdom; ^6^ University College London, Electrochemical Innovation Lab Department of Chemical Engineering London United Kingdom

**Keywords:** beam tracking, gratings, microfluidics, x‐ray masks, x‐ray phase contrast imaging

## Abstract

Fabrication of X‐ray gratings has surged in the last two decades thanks to their key role in X‐ray Phase Contrast Imaging, an imaging technique able to boost X‐ray sensitivity to detect otherwise invisible details. These high aspect ratio devices are usually fabricated by complex, costly, multi‐step processes that limit their size and production throughput. These steps commonly involve UV or X‐ray lithography, semiconductor selective etching, and high‐Z metal plating, usually Au, which require expensive tools and materials. Here we present a proof‐of‐concept fabrication via soft lithography and Hg infusion of microfluidic X‐ray absorption gratings and their performance in biomedical imaging. In this work, we demonstrate gratings with a thickness of 21 μm, made of 10 μm‐wide lamellae and 59 μm‐wide Hg‐filled channels. While these initial parameters were chosen to validate the infusion process, significantly higher aspect ratios are attainable using similar, established microfluidic fabrication techniques. Such fabrication technique requires fewer, less expensive, and more scalable processes using alternative and more sustainable materials, while showing comparable visibility with their conventional Au‐based, solid equivalent. Our results constitute a promising shift in X‐ray optics fabrication that could significantly lower barriers to commercialization and accelerate the practical deployment of X‐ray Phase Contrast Imaging.

## Graphical abstract

This work establishes a new protocol for the fabrication of X‐ray gratings that exploits the advantages of microfluidics and enables a more inexpensive, faster, and scalable production process. These X‐ray devices are fundamental for implementing X‐ray phase contrast imaging (XPCI) in laboratory and clinical settings, yet their costly and complex fabrication has hindered the broad adoption of this technique. Since XPCI has been shown to improve contrast in many relevant clinical and industrial contexts, this fabrication procedure could potentially pave the way for the translation of XPCI into real‐world applications.

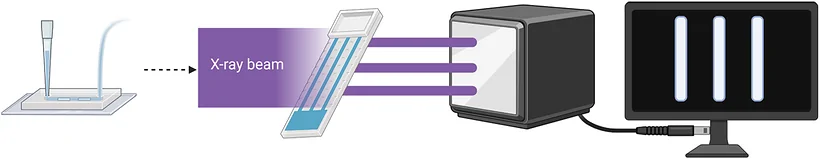

## 1 Introduction

X‐ray imaging (XRI) has profoundly impacted modern life over the past century, with applications ranging from medical diagnostics and non‐destructive industrial testing to employment in scientific research [1, 2, 3]. As it is conventionally based on attenuation contrast, thus detecting details with different attenuation coefficient than the background, XRI falls short in detecting low‐Z materials (e.g., organic structures). Thus X‐ray Phase Contrast imaging (XPCI) has attracted significant attention in the last three decades as an alternative imaging method due to its higher sensitivity to low‐Z features [4].

In the X‐ray regime, most elements' refractive index n=δ+iβ is characterized by a real part δ exceeding the imaginary part β by orders of magnitude. This means that higher image contrast can in principle be obtained from the detection of an X‐ray beam's refraction induced by an object rather than its attenuation. By exploiting this mechanism, XPCI enhances the sensitivity of conventional XRI, enabling the visualization of otherwise low‐contrast structures, such as soft tissues, early‐stage tumors, pulmonary emphysema, microfractures in composite materials, and explosives in security scans [5, 6, 7, 8].

Despite the significant improvement in contrast, XPCI is limited by its requirement for coherent beams typically not attainable with the X‐ray sources used in industry or hospitals. To circumvent this requirement, a series of imaging techniques have been devised based on the modulation and analysis of not necessarily coherent beams. This was achieved by implementing high‐aspect ratio (AR = lamellae's height/width > 10) gratings in the imaging set‐up to modulate the beam intensity and/or phase. The high‐AR gratings can be optimized to either selectively mask or impart a phase shift to the X‐ray beam by tuning the thicknesses and widths of their transparent lamellae and highly absorbing/phase shifting septa, being referred to as absorption grating and phase grating, respectively. Regardless, they both require high AR due to the the incoming X‐ray photons' high penetration depth, and very fine lateral dimensions (1–100 μm) due to the small refraction angles induced at these energies (a few μrad). Such extreme critical dimensions significantly hinder their fabrication, which was enabled only in the last two decades by a series of technological improvements and novel fabrication processes, which ultimately allowed the translation of XPCI to laboratory and clinical set‐ups [9, 10].

Building on seminal gratings work,[11, 12, 13] the development of high‐AR, metallic‐supported gratings has been instrumental in overcoming the coherence limitations of laboratory X‐ray sources [9]. Since then, a lot of effort has been made to increase the attainable AR up to ∼ 40 [14, 15, 16], thickness up to ∼ 500 μm [17], and to reduce their periods down to sub‐μm range to accommodate the requirements of different phase‐based imaging approaches [14, 15]. Nevertheless, the fabrication paradigm for higher‐AR gratings has not moved away from the lithography‐etching‐plating pipeline, with the exception of LIGA i.e., lithography, plating, molding to replicate, which however requires highly coherent and brilliant synchrotron radiation or exposures of multiple days with conventional tubes [18, 19, 20]. In both pipelines lithography represents the bottleneck for the attainable period and aperture, as the photoresist (PR) patterning is limited by optical constraints when PRs are exposed to UV e.g., wavelength, numerical aperture, PR optical properties. E‐beam lithography and Laser Interference Lithography have been used to reduce the attainable periods to the sub‐μm range, at cost of significantly longer processing time for the former and smaller exposure area for the latter [14, 15]. As higher ARs are preferred, PRs are often employed as etching masks for the underlying substrate (mostly Si) [9, 16, 17, 21, 22]. The most popular methods for deep etching of Si are deep reactive ion etching (DRIE) and metal assisted chemical etching (MACE), a dry, plasma‐induced and a wet method, respectively. The former can reach high etching depths thanks to the multi‐cycle Bosch process and the careful optimization of passivation and etching steps [23]. The latter instead exploits Si chemical oxidation and dissolution in solutions comprising an oxidant, usually $H_{2}O$ 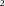, and HF as an etchant, which constitutes a significant health hazard [24]. This reaction chain is usually very slow though it can be catalyzed by patterning the exposed Si substrate with noble metals such as Au and Pt, driving the reaction downward [25]. Finally, Au is the preferred material as beam absorber/shifter thanks to its high density and refractive index combined with its high reduction potential, which makes it relatively easy to plate onto a conductive substrate [26]. In the X‐ray grating context, the substrate is either an etched semiconductor (i.e., Si) or developed PR if a LIGA approach is followed. They both do not favor Au plating due to their high resistivity, therefore requiring an additional step of deposition of a seed layer. Atomic layer deposition (ALD) represents the gold standard for conformal seed layer deposition [14, 27], although physical sputtering is sometimes preferred due to its lower cost and overall duration [15, 17]. Recently, low‐resistance Si has been used to enable seedless plating by maintaining a low plating rate and a positive tapering of the sidewalls [28]. In MACE and LIGA, a bottom‐up growth can be obtained using the metal catalyst or a preliminarily deposited seed layer, respectively [19, 22].

Fabrication costs increase and success yield decrease with the number of processes required. Expensive materials (e.g., Au and other noble metals) and sophisticated equipment like DRIE, PVD, and ALD tools increase costs too, which would also grow proportionally with grating size, as these processes would take longer and/or require larger and more expensive equipment. The high costs and fabrication hindrances are nowadays one of the main obstacles to the widespread use of XPCI, so that low‐cost modulators such as abrasive papers have been already tested, enabling easier systems at the cost of smaller pixel size and larger coherence when compared with grating‐based methods [29]. This paper shows how alternative processes and materials can provide viable grating fabrication protocols. A new grating design is presented, employing microfluidic polydimethylsiloxane (PDMS) channels patterned via soft lithography as a substrate and filling the channels with highly absorbing fluid Hg. Hg has comparable atomic number and mass density as Au, making it a viable alternative as absorbing material in the channels (here having the same role as the septa in traditional masks) with the benefit of being flown in the PDMS microfluidic channels via simple pumping. The use of soft lithography for the fabrication of PDMS channels, despite currently reaching lower ARs in this proof‐of‐concept work, has a series of benefits over the conventional Si lithography and etching: it is more easily scalable and recyclable, it is inexpensive as it does not require intensive use of specialized equipment and allows for multiple replication of each individual mold [30, 31]. Soft lithography generally involves a lithographically fabricated mold and a liquid precursor of an elastomer cast on it, curing and solidifying on the master mold, finally taking its complementary structure. The limitations therefore stem from the lithographic method used to fabricate the mold (in common with the previously listed grating fabrication methods) and the elastomer's mechanical properties [32]. However, PDMS microfluidic devices have been fabricated via soft lithography with a wide range of dimensions (from nanometric channels to multiple hundreds of micrometers), ARs (up to 20), and designs [33, 34, 35, 36]. More X‐ray optics such as phase‐shifting gratings for Talbot‐based interferometry, with periods usually lower than 5 μm, can therefore be fabricated following a microfluidic protocol. It also potentially enables the fabrication of flexible and curved gratings, which is currently limited by the Si and Au stiffness to large curvature radii [37]. Flexible gratings would accommodate conical beams from conventional X‐ray sources, therefore extending the imaging field of view (FOV) with resulting extended applicability in industrial and clinical set‐ups. To this date, larger FOV (∼ 38.5 cm × 2.5 cm) in the first prototypes of human‐scale XPCI‐CT scans has been reached by grating tiling, instead thus suffering from regions of affected contrast in the areas between gratings [38].

In this work, we present the fabrication and experimental validation of such microfluidic gratings, which are successfully employed in a Beam Tracking (BT) XPCI setup demonstrating robust performance and yielding high‐quality, quantitatively accurate phase‐contrast images [39, 40]. The choice of a BT approach, where a single absorption grating is used to selectively mask the X‐ray beam, allowed us to: (1) individually test our gratings performance (which in the context of refractive imaging techniques such as BT are usually referred to as ‘mask’, a term that will also be used in this work when appropriate), (2) efficiently image biological samples obtaining higher contrast in phase‐based images compared to attenuation‐based, while (3) simultaneously relaxing the coherence requirements by using a 70 μm focal spot, uncollimated and fully polychromatic x‐ray source. This alternative X‐ray grating fabrication protocol could be instrumental for translating XPCI into real‐world applications by reducing its entry cost for clinics and hospitals. Despite not yet matching the best achievable results from gold standard fabrication protocols (e.g., DRIE + superconformal growth, or LIGA), microfluidic devices have been reportedly fabricated with critical dimensions and aspect ratios comparable to the requirements of several X‐ray optical elements for XPCI [38, 41]. As XRI remains the most used diagnostic imaging techniques, unlocking XPCI's potential could have an impact on millions of lives worldwide [42].

## 2 Results

**FIGURE 1:**
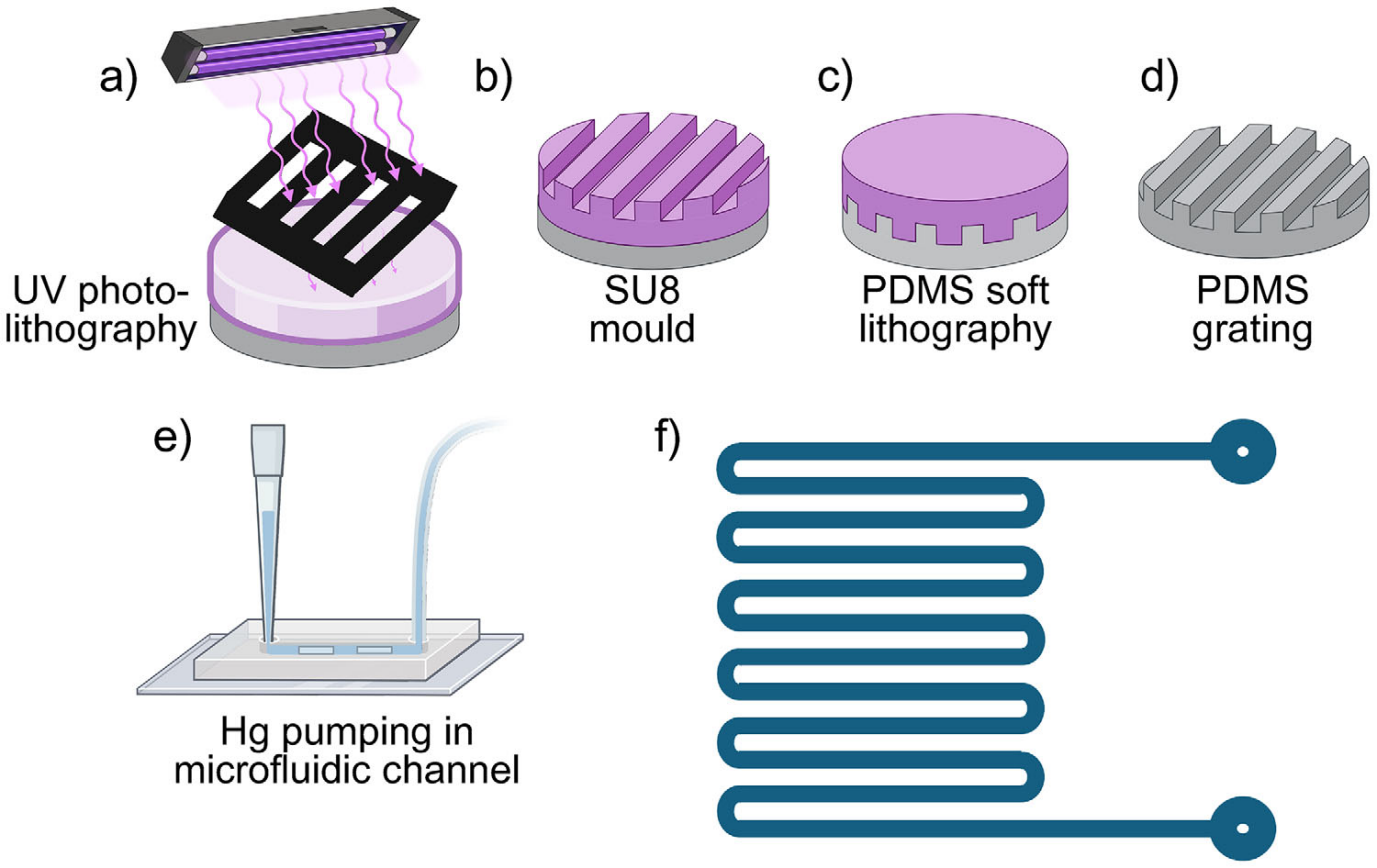
Fabrication process. (a) SU8 layer exposure to UV‐photolithography using a photomask, resulting in a (b) patterned SU8 mold. (c) Replication on polydimethylsiloxane (PDMS) via soft‐lithography and (d) demolding. (e) Chip fabrication via bonding with glass and Hg pumping. (f) Microfluidic chip scheme of the design: a serpentine with two infusion/extraction channels to pump Hg in/out. Created in BioRender. Rossi, A. (2026) https://BioRender.com/8ux17a5.

A scheme of the fabrication process used in this work is shown in Figure 1. The fabrication consisted of a common soft lithography protocol (Figure 1a–d) using a SU‐8 patterned mold transferred onto a PDMS covalently bonded to a glass slide, followed by Hg pumping inside the obtained channels via a peristaltic pump (Figure 1e). The design chosen for the microfluidic channel is schematized in Figure 1f: a serpentine with two infusion/extraction channels to pump in the Hg and pump out the excess, respectively. The microfluidic chip was finally employed for imaging exploiting the Hg channels beam splitting properties. The fabrication processes and the materials used are discussed in more detail in the Experimental Section.

### 2.1 Microfluidics Channel

The microfluidic device was designed with a periodicity of 79 μm, with channel widths of 69 μm and an inter‐channel spacing of 10 μm. These dimensions were chosen based on the prospective BT imaging system the devices would be tested in. Fabrication was carried out using standard UV and soft lithography processes. UV lithography was employed to produce a rigid polymeric mold, which served as a template for replication in PDMS (Figure 1a–c). A two‐dimensional CAD design containing the entire device layout was created using Rhinoceros CAD software and subsequently produced via photoplotting by Fine Line Imaging, Inc (Colorado, USA). The design consists of a continuous serpentine‐shaped channel with a single inlet and outlet, connected to the serpentine via several‐mm‐long channels (see Figure 1e). The serpentine design also enables eliminating transversal connections (often referred to as bridges) used as support in lithographic techniques, which can cause artifacts at the imaging stage [43].

**FIGURE 2:**
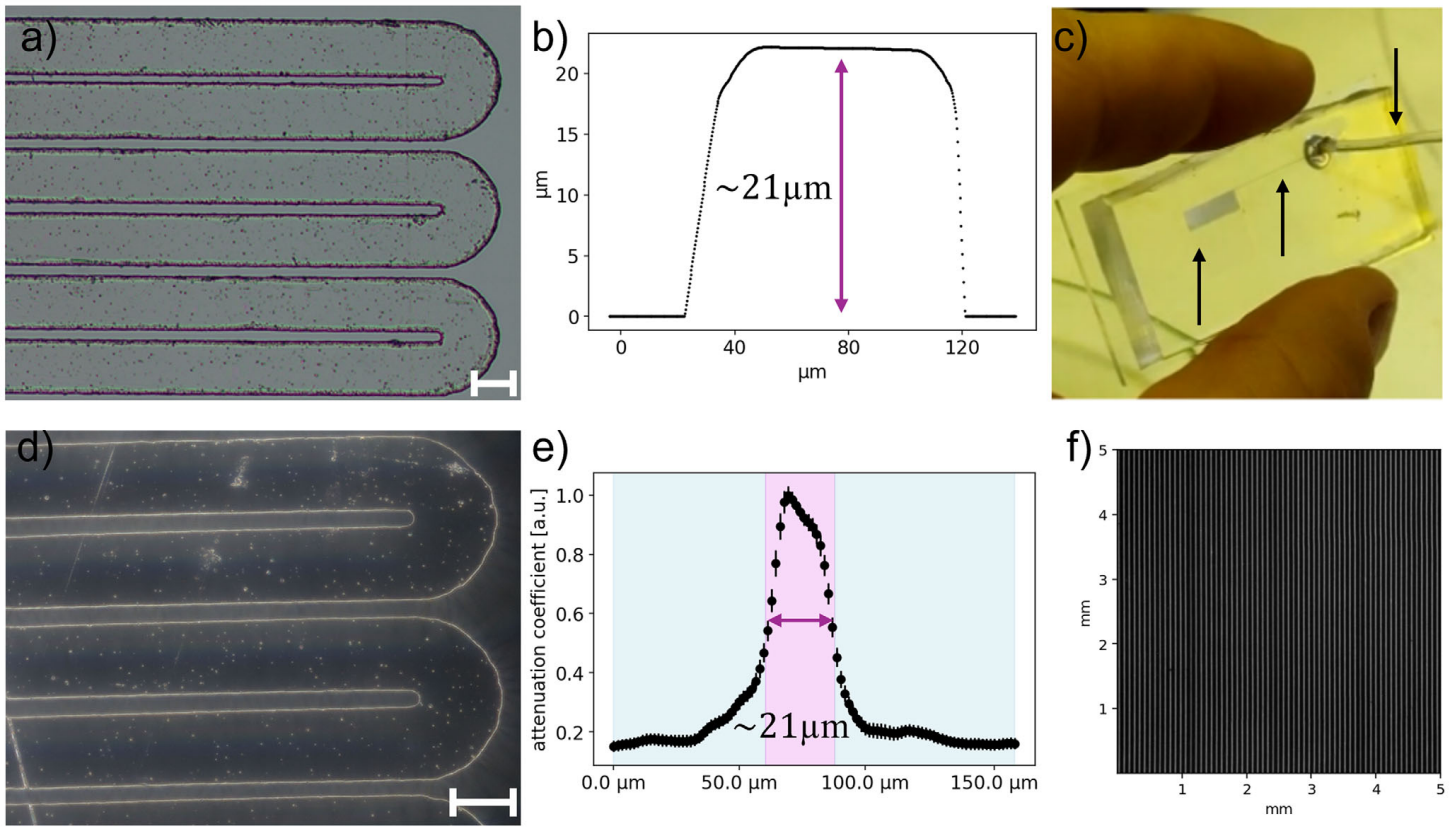
(a) Optical microscopy of the SU‐8 mold. Scale‐bar: 50 μm. (b) Profilometry measurement of SU‐8 mold inlet channel. The protrusive ∼ 21 μm channel is later imprinted in the PDMS chip. (c) Microfluidic chip during Hg pumping from the inlet to the serpentine through the loading channel (highlighted by black arrows). (d) Optical microscopy of the Hg‐filled mask. Scale‐bar: 50 μm. (e) Attenuation coefficient profile of Hg channel (light purple shading) sandwiched between PDMS and glass (light blue shading), averaged over 1000 CT slices (of which two examples are shown in Figure S1) and 10 channels. (f) Transmission‐based radiography across multiple mm of the mask (pixel size = 5 μm).

An ordinary microscope glass slide was used as a substrate for the microfluidic chip. The glass was plasma treated and coated with a 25 μm‐thick layer of SU8‐2050, a negative epoxy‐based PR, via doctor blade coating. The coating was patterned using a mask aligner equipped with a 365 nm LED source at a dose of 160 mJ/${\mathrm{cm}}^{2}$ and the photomask produce by Fine Line Imaging, Inc. The coated substrate was baked before (soft baking) and after (post‐exposure baking) exposure. Final development was carried out by immersing the sample in developer under gentle swirling for 6 min. The resulting SU8‐mold was inspected using an optical microscope (Zeiss Axiolab 5 microscope) and a profilometer (Dektak 8, Veeco), and the results are shown in Figure 2a,b, respectively. The former showed a periodicity of 79 μm and spacings of 10 μm as desired, and the profilometry across the channels detected a final thickness of ∼ 21 μm. The thickness reduction from the nominal 25 μm is attributed to solvent evaporation during processing [44].

This mold was then used to fabricate a PDMS replica using Sylgard 184 at a 10:1 elastomer/curing agent ratio. The mixture was thoroughly mixed, degassed, poured onto the mold, and thermally cured for 90 min. To facilitate the final demolding, the mold was previously treated with HMDS, a hydrophobic surfactant that minimizes the interaction with the PDMS‐molded replica, by dip coating. After demolding, a microscope glass slide was placed on the PDMS‐replica, and the assembly was exposed to plasma in air for 20 s to functionalize the surface and enhance adhesion. This resulted in a strong, covalent bond between the PDMS channels and the glass slide, which ensured final encapsulation of Hg in the channels. The Hg was finally introduced in the channel using a peristaltic pump, as shown in Figure 2c, where the Hg flowing inside the serpentine can be observed. The three arrows in Figure 2c highlight (from left to right) the serpentine‐channel being filled, the loading channel connecting the serpentine and the inlet through which the Hg is enabled to flow from the peristaltic pump.

### 2.2 Mask Characterization

The filled mask was characterized via optical microscopy and X‐ray transmission imaging, both in planar and CT scans. Images taken at the optical microscope reveal features in agreement with the ones found in the mold (Figure 2d), proving an adequate mold replication and the feature preservation upon Hg filling. In both Figure 2a,d, the curved regions of the serpentine are observed. While the optical image reveals some surface‐level defects and minor damage, these are not expected to affect mask functionality and are in fact absent in the X‐ray images, which confirms the presence of continuous, Hg‐filled channels. Notably, both images were acquired over two years after fabrication, underscoring the long‐term structural integrity of the device. This stability highlights the effectiveness of the PDMS‐glass chip, which encapsulates the Hg and provides both mechanical robustness and a critical barrier for toxicity containment.

To confirm the complete filling of the channels with Hg a μ‐CT scan was performed. This choice was preferred over other destructive methods such as electron microscopy to preserve mask integrity and avoid Hg spillage. The non‐destructive μ‐CT scan enabled us to fully resolve the channels across multiple millimeters through an effective pixel size of approximately 1.6 μm; details on the used μ‐CT system and data reconstruction are provided in the Experimental Section. The Hg channels were unsurprisingly characterized by a higher attenuation coefficient compared to the surrounding PDMS regions, as shown in Figure 2e, allowing an easy discrimination between Hg and PDMS or glass regions, respectively highlighted in light purple and blue in the plot. Despite some variation across the channel profiles, the thickness of the Hg channels amounts to 21 μm ± 2 μm, matching well the SU‐8 mold's depth shown in Figure 2b. Two representative CT slices used to measure the Hg depth are presented in Figure S1. This result proves (1) the full transfer of the serpentine features to the PMDS replica, and (2) the complete filling of the channels. Finally, X‐ray transmission radiography was performed on the mask to investigate its performance with imaging systems at lower resolution. One of these images is shown in Figure 2f, highlighting good homogeneity of the channels over multiple millimeters. The mask's testing and employment in XPCI setups is reported in the next sections.

### 2.3 Beam Tracking Imaging

**FIGURE 3:**
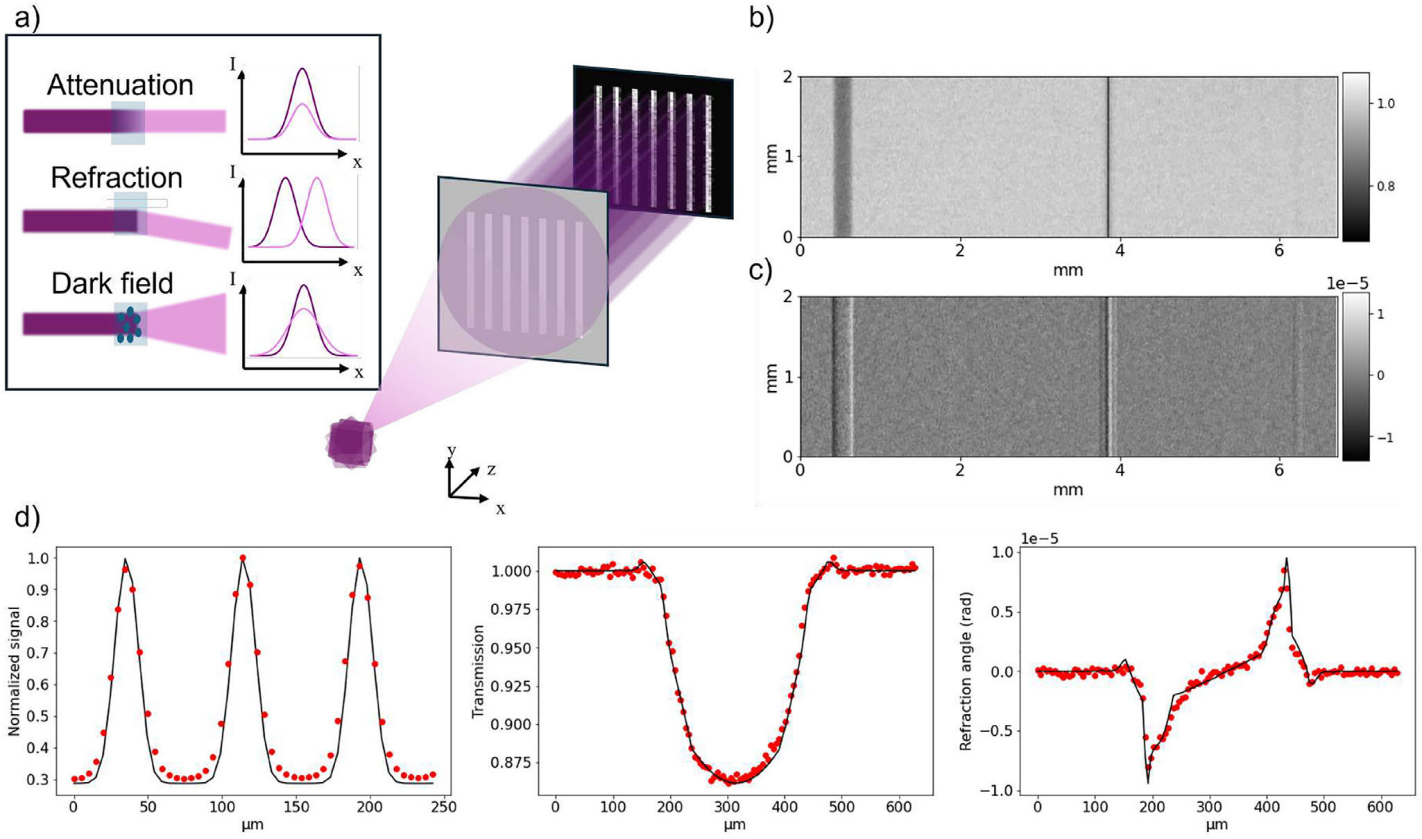
(a) Scheme of a BT imaging setup. The purple source emits a conical beam split by the grey mask into individual, well‐separated beamlets resolved by the black detector at the back. In presence of a sample, the beamlets are perturbed, allowing for analysis and retrieval. The sample's attenuation, refraction, and scattering effects on the beamlets are reported in the inset on the left. (b) The attenuation‐based radiography of three wires i.e., (from left to right) sapphire, boron with a W‐core, and nylon. (c) Corresponding refraction‐based image, highlighting the edges and the nylon wire, almost invisible in the attenuation‐based image. (d) Comparison between experimental data (red points) and simulated values (black profiles). The normalized profile on the left represent three unperturbed beamlets. The plots in the middle and the right show transmission and refraction through a 250 μm wide sapphire wire, averaged vertically over 100 pixels to compensate for microscopic wire deformation or misalignment.

BT is a single‐mask phase‐based imaging approach, typically simpler to implement than the other grating‐based techniques, as no particular alignment is required and only one element is required to fabricate. The absorption grating selectively masks the X‐ray beam creating individual beamlets whose sample‐induced attenuation, refraction, and scattering can be directly analyzed by a detector with pixel size smaller than the beamlet's width (Figure 3a, individual beamlets intensity shown in dark purple and pink for ‘reference’ and ‘object’ curves, respectively) by gaussian interpolation or statistical moments [40]. These retrieval algorithms are discussed in more details in the Experimental Section.

Due to their simple geometry and homogeneous structure, cylindrical wires are often used as benchmark for phase‐based imaging setups, as they allow for a straightforward comparison against simulated results [45, 46, 47]. Three different wires (sapphire, boron with a W‐core, and nylon) have been placed immediately downstream of the mask as shown in Figure 3a and imaged using a rotating anode X‐ray source. The wires (and all the X‐ray images shown in this work) were imaged using a polychromatic 40 keV beam generated by a Mo anode with a mean delivered energy of approximately 21 kV. The exposure time was set to 1 s, and the remaining experimental parameters (detector and source parameters, distances, etc.) can be found in the Experimental section. The retrieved attenuation and refraction images of the wires are shown in Figure 3b,c. The refraction contrast is consistently higher allowing to detect wires otherwise barely visible in attenuation, such as the thin nylon wire on the right in the two images. The different contrast channels were retrieved with a moment‐based algorithm [48]. The same system was then numerically simulated in a wave‐optics based approach using the mask dimensions found during characterization. The same retrieval method was used to separate the different signals (transmission, refraction) for the simulated data and compared with the experimental ones, showing very good agreement as visible in Figure 3d. The figure presents, from left to right, the comparison of normalized intensity profiles across three mask periods unaffected by the sample, followed by the transmission and refraction profile of a sapphire wire, respectively; comparisons for the other wires can be found in Figure S2. The left plot in Figure 3d shows three well separated beamlets, evidence of an efficient masking of the beam by the microfluidic device. The transmitted intensity through the absorbing Hg channels is roughly 30%. Although, unlike in grating interferometry, the visibility does not affect the phase sensitivity in the beam tracking approach used here in the same way, this would correspond to a visibility of approximately 54%. A thicker channel would reduce the baseline further, increasing the beamlet visibility and ultimately the phase sensitivity. Doubling the thickness would lead to a 10% transmission, with a consequent increase in sensitivity. The refraction profile (right plot) shows abrupt changes of intensity, rather than a smooth gradient, due to a combination of blurring due to finite source size and detector's point‐spread function, discretization of pixel sampling, and the retrieval method [39].

Maximizing the absorbing septa's stopping efficiency has been a persistent issue in X‐ray imaging, as Au deposits via plating commonly suffer from sub‐bulk densities resulting in lower visibility [14, 15, 49]. This issue is worsened when alternative plating methods such as metallic (e.g., W, Au, and Pt) centrifugal depositions are used and an even lower absorption efficiency is reached [50, 51, 52]. In this work we did not encounter any of these issues due to the Hg fluid physical state and bulk‐like density maintained in the microfluidic channels, which enabled the maximum absorption given the aspect ratio obtained. Our interpretation is that the Hg fluid state minimizes scattering and visibility degradation due to its inherent lack of granularity, which has instead been reported for Au‐plated and metallic centrifugal deposits [14, 16, 21, 50]. While visibility degradation can arise from external factors, such as imaging system vibrations, the use of a microscopically homogeneous material for the grating septa provides an advantage in terms of eliminating coherent scatter from the grating septa themselves, which can reduce visibility. Eliminating granularity has also an advantage in terms of x‐ray absorption provided by a given thickness of a certain material.

### 2.4 μ‐CT Scan

**FIGURE 4:**
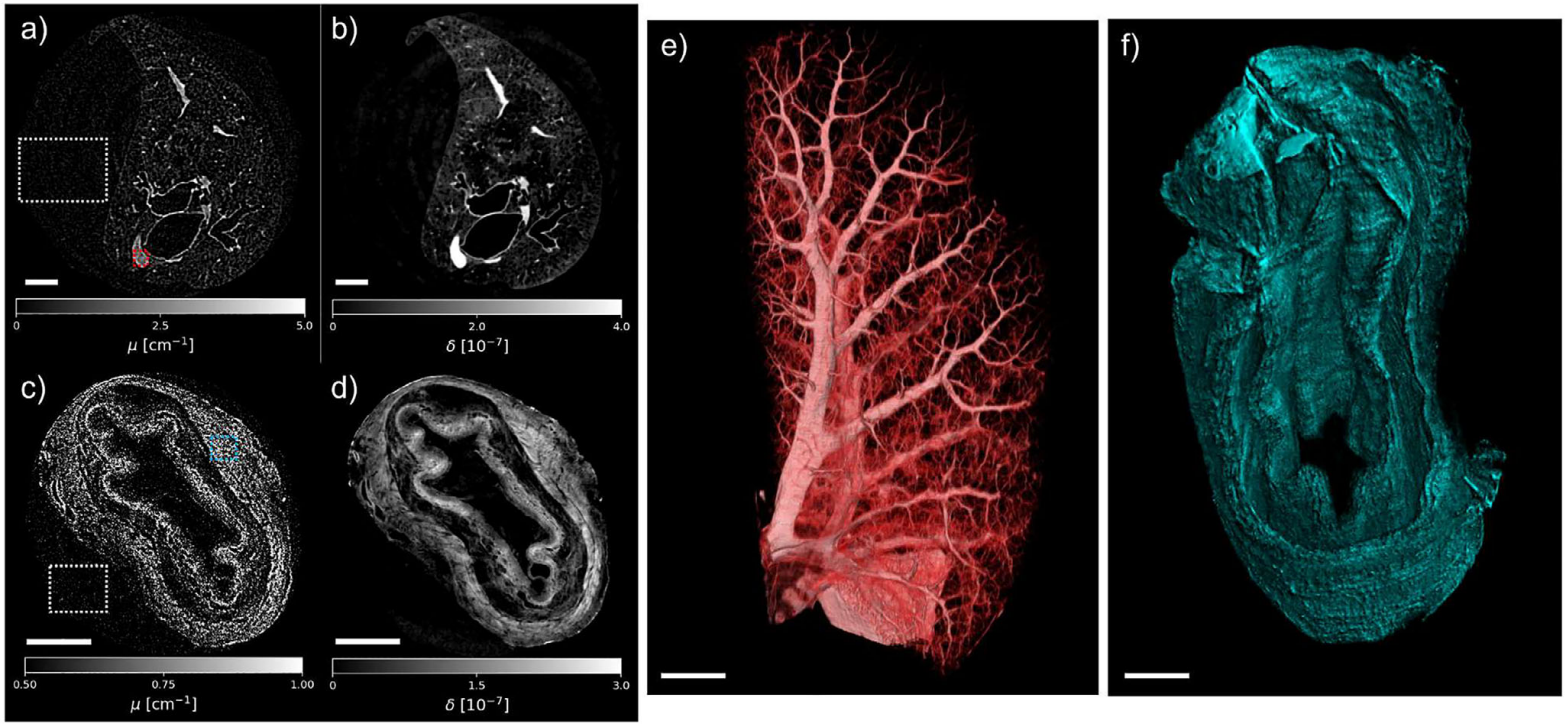
(a,b) Cross‐sectional slices of the mouse lung obtained from attenuation‐ and phase‐based reconstructions, respectively. (c,d) Corresponding attenuation‐ and phase‐based slices of the piglet esophagus. Scale‐bars: 2 mm. CNRs for the features highlighted in the red and cyan boxes (a, c) were calculated relative to the background regions indicated by the dashed white boxes. The same ROIs were used to extract the CNR from the images in panels (b) and (d) for consistency. To improve statistics, all ROIs were averaged across 5 slices. (e,f) Volumetric reconstruction of the lung and esophagus, respectively. Scale‐bar: 1 mm.

The same BT set‐up used for the wires was employed to perform μ‐CT scans of two biological samples: a mouse lung (Figure 4a,b) and a piglet esophagus (Figure 4c,d). Samples were obtained from animals sacrificed for other, ethically approved studies, i.e., no animals were sacrificed specifically for this study. Indeed, images of the same specimens obtained with a different system can be found in Modregger et al. [53] and Savvidis et al. [54], respectively. The samples had been critically point dried and safely stored to minimize significant morphological variations. The same references also provide full details on the sample preparation methods. The samples were rotated over $360^{\circ }$ in 1200 equally spaced projections maintaining a 1s‐long exposure for each projection, dithering (i.e., laterally shifting) the samples across a mask period in 8 and 16 steps for the lung and the esophagus, respectively. As can already be appreciated in the results in Figure 3, these masks yield phase‐contrast images with significantly higher contrast‐to‐noise ratios (CNR) compared to their attenuation counterparts. Specifically, the transition from attenuation to phase‐based imaging results in a CNR increase, calculated within the regions of interest (ROIs) indicated in Figure 4, from 5.3 ± 1.4 to 24.2 ± 1.3 for the lung (Figure 4a,b) and from 1.6 ± 1.2 to 10.6 ± 2.4 for the esophagus (Figure 4c,d). As a result, a multitude of details are made visible in the phase‐based cross‐sectional slices (Figure 4b,d) of both samples compared to their attenuation‐based ones (Figure 4a,c). The phase‐based image of the mouse lung highlights the bronchi, sharpening their edges and unveiling some left undetected by the attenuation‐based counterpart. Similarly, the phase‐based image of the piglet esophagus shows much sharper edges and a series of details completely undetected by the attenuation‐based image in the epithelium and the submucosa region. The system's phase sensitivity can be further increased (i.e., leading to the same contrast values, but a higher CNR ratio for the same exposure time [55]) by increasing the microfluidic grating's aspect ratio, which remains a goal for the future.

This image quality however provided a high enough contrast to render the imaged biological samples in 3D volumes using commercial softwares (see Experimental Section). Figure 4e shows a 3D volume render of the lung, where several generations of airways are clearly resolved and displayed in a red‐scale. Figure 4f displays the esophagus volume with a central cubic section exposed to reveal the internal submucosa structure, otherwise covered by the external walls, rendered in cyan.

## 3 Discussion

This study demonstrates the feasibility and discusses the prospective advantages of employing microfluidic X‐ray masks for XPCI. Their effectiveness for phase‐based imaging was validated in a BT setup, enabling quantitative refraction‐based image acquisition with enhanced sensitivity to low‐Z samples (i.e., wires, mouse lung, and esophagus) compared to the conventional attenuation‐based counterpart. The deployment of these microfluidic masks is not limited to BT however, as several imaging modalities use absorption masks, thus promising a wide applicability across the field.

This fabrication method for microfluidic X‐ray gratings is compatible with a variety of materials and processes, opening a yet untapped research path in XPCI. In the work described here, soft lithography with PDMS was used to replicate the lamellar grating structure, and Hg was introduced into the channels via peristaltic pumping to create the X‐ray absorbing septa. However, alternative strategies can be employed to achieve similar grating functionality. First, hard‐PDMS has proven to increase the attainable AR by soft lithography compared to conventional PDMS used in this work [32]. The lamellae could also be patterned directly in PR using standard UV or X‐ray lithographic techniques, providing higher ARs. Epoxy‐based PRs like SU8 can now commonly achieve few hundreds‐micrometer high gratings [56] and also be used as molds for PDMS high‐AR (AR > 20) structures [35]. Other replication techniques include hot embossing and nanoimprint lithography, allowing direct transfer of high‐AR features into thermoplastic materials, reaching sub‐micrometer precision [57, 58, 59].

Similarly, other high‐Z contrast agents such as iodine‐based solutions or metal alloys (e.g., Rose metal) may replace Hg to serve as the absorbing medium, particularly in situations where toxicity is a concern. This may lead to a reduction in visibility, but also e.g. exploit the increased attenuation at the K‐edge energies of specific materials. It is important to emphasize that the amount of Hg required to fill meter‐long microchannels is on the order of tens of milligrams, allowing for low exposure to Hg within most regulations (e.g., OSHA, NIOSH, EH40/2005 [60, 61]). The pumping was performed under controlled ventilation to further mitigate the risk of vapor inhalation. Finally, the long‐term integrity of the glass/PDMS chip developed in this work, which showed no leakage or damage over multiple years, demonstrates that Hg‐filled microfluidic systems can be safely used without risk of inhalation or leakage, as long as sealing is maintained.

While Hg peristaltic pumping offers reliable and controllable loading of the absorber medium, alternative approaches such as Hg porosimetry may be explored for applications requiring pressure‐tuned filling. Such method would also allow an efficient fluid recovery from the microchannels and reuse, aligning with the principles of circular economy and safer toxicity management, requiring only a moderate applied pressure according to Young‐Laplace law (∼ 0.1 bar) [62]. Additionally, the PDMS matrix (and protective layer) itself is recyclable under certain conditions [63], further supporting the sustainability of this fabrication approach, while ensuring year‐long durability and safe handling. The flexibility of this method, across both structural and absorbing materials, makes it adaptable to various imaging requirements and system constraints. Thus, the technique is not limited to the PDMS/Hg implementation described here but is broadly extensible to other material combinations and filling strategies.

More generally, as the interest in flexible X‐ray imaging systems grows [64], the need for adaptable and mechanically compliant optical components becomes increasingly important. Microfluidic gratings fabricated in elastomeric materials, such as PDMS offer the compelling advantage of conforming to curved geometries without significant degradation. Unlike rigid gratings, which are limited by brittleness and can fracture under bending [37], these soft gratings can potentially withstand much larger radii of curvature, making them well‐suited for integration into highly divergent X‐ray imaging beams.

## 4 Conclusion

In conclusion, this work demonstrates a simple fabrication protocol for X‐ray microfluidic gratings using soft lithography and peristaltic pumping. The prototype device described in this paper unlocked phase‐based images with higher contrast compared to their attenuation‐based equivalent despite the relatively low AR achieved. The translation to higher ARs and fully flexible materials will be the object of future work. We believe that this new approach will play a pivotal role in translating XPCI into industrial and clinical settings. The main reason lies in the ease of inexpensively scaling up this approach, which relies on a two‐step process rather than the complex, multi‐step workflows typically required. Currently, the cost of fabricating a few ${\mathrm{cm}}^{2}$ of grating for XPCI remains in the range of the thousands of dollars, with even higher investments needed when developing or optimizing new processes. By contrast, our method significantly lowers both the entry barrier and per‐unit cost (up to two orders of magnitude lower), allowing a broader adoption, rapid prototyping, and faster translation to practical imaging environments.

## 5 Experimental Section

### 5.1 Fabrication Process

The microfluidic chip was fabricated via two lithographic‐step processes: mold patterning via UV lithography and microfluidic channel replication via soft lithography. The rigid mold was fabricated in SU8‐2050 coated on glass by doctor blade technique. The coated substrate was soft baked on a hot plate (Calctec, Italy) in two steps to reduce residual stress in the film. First, it was baked at 65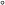 C for 2 min and 30 s, followed by a temperature ramp of 10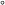 C/min to 95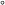 C and baking for 6 min. After cooling to room temperature, the SU8 was exposed by mask aligner (UV‐KUB 2, Kloé, France) at 365 nm at with a dose of 160 mJ/cm using the serpentine‐shaped photomask produced by Fine Line Imaging, Inc. The post‐exposure bake was performed in two steps too on the same hotplate as the soft baking. It consisted of an initial baking at 65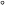 C for 90 s followed by a 10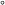 C/min ramp to 95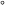 C and 6 min‐long bake. Development was carried out by immersing the sample in SU8 developer under gentle manual agitation for 6 min. The PDMS‐replica was prepared by mixing Sylgard 184 at a 10:1 elastomer/curing agent ratio, followed by degassing and curing at 70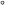 C for 90 min. The SU8‐mold was hydrophobically functionalized by dip‐coating in a solution of HMDS dissolved in toluene (5% molar concentration) for 5 s. The finalised microfluidic chip was filled with Hg by pumping using a peristaltic pump (Dolomite Mitos System, Unchained Labs, USA) connected to the loading channel in the chip via a Teflon tube. The outlet channel in the chip was also connected to a tube to accommodate overflow and collect possible Hg spilling.

### 5.2 X‐Ray Imaging

The BT images (Figures 3b–d and 4) and the mask X‐ray characterization (Figure 2c) have been acquired on a customized imaging set‐up using equipment available at the UCL Advanced X‐Ray Imaging (AXIm) Labs following the scheme in Figure 3a. A Rigaku M007 X‐ray source (Rigaku Corporation, Tokyo, Japan) with a rotating Mo target (70 μm focal spot, 30 mA) provided a polychromatic beam at 40 kVp. A Hamamatsu (Hamamatsu, Shizuoka, Japan) C12849‐111U detector with 10 μm‐thick GdO scintillator and 6.5×6.5 μm 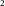 pixel size was used, enabling directly resolving the beamlets created by the pre‐sample mask as required by the BT technique. Our mask was placed at *d* 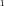 = 0.54 m from the source and *d* 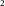 = 0.17 m from the detector, amounting to a relatively compact set‐up of total length of *d* 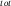 = $d_{1}$ + *d* 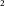 = 0.71 m. Such distances resulted in a mask magnification on the detector plane of *M* = $\frac{d_{tot}}{d_{1}}$ ≈ 1.31. With the mask period being 79 μm, the individual beamlets were well separated, with one mask period covering approximately 16 pixels on the detector plane. The samples were placed immediately downstream the mask to maximize the obtained refraction angles and scanned across one mask period in 8 (for the lung) and 16 (for the esophagus and the wires) dithering steps along the horizonal direction *x* in Figure 3a. The sapphire, boron, and nylon wires (Goodfellows) are 250, 200, and 100 μm in diameter, respectively. The W‐core in the second wire has a 10 μm diameter.

For the CT scans, the samples were rotated along the vertical axis (*y*‐axis in Figure 3a) over 360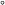 in 1200 evenly spaced projections. All the images were taken with 1s‐long exposure times.

The mask X‐ray μ‐CT scan was performed using a Zeiss Xradia 620 Versa with a 4X magnification lens resulting in a (magnified) pixel size of approximately 1.6 μm. The tube voltage was set at 160 kVp with a power of 17 W. Across the 2001 CT‐projections taken, a High‐Aspect‐Ratio‐Tomography (HART) strategy was adopted. HART is designed to increase the exposure time of a range of projections corresponding to the angles with the longest photon path through the sample. These projections, which in a planar sample like our mask correspond to the range of angles along which the mask's longer side is almost parallel to the beam, suffer from photon starvation resulting in artifacts in the image. Therefore, the HART strategy used here quadrupled the exposure time (20 s) on the appropriate range of angles, effectively suppressing these artifacts. The object reconstruction was performed using Zeiss proprietary software Advanced Reconstruction Toolbox.

### 5.3 Retrieval

Two different retrieval methods were used in this work: the moment‐based and the gaussian fitting methods [53, 65]. The former is simpler and faster than the latter, being well suited for very simple samples such as the wires. The more complex biological samples required a more sophisticated retrieval process i.e., the gaussian fitting method. The moment retrieval is based on the computation of the zeroth, first, and second statistical moment for each beamlet *I* over its length *l*, corresponding to approximately 16 pixels. These values represent the beamlet's area ($M_{0}$), center ($M_{1}$), and variance ($M_{2}$) as in the equations below:

(1a) $$\begin{array}{cc} M_{0} & =\int_{0}^{l}I\left(x\right)\;dx \end{array}$$

(1b) $$\begin{array}{cc} M_{1} & =\frac{1}{M_{0}}\int_{0}^{l}x\cdot I\left(x\right)\;dx \end{array}$$

(1c) $$\begin{array}{cc} M_{2} & =\frac{1}{M_{0}}\int_{0}^{l}{\left(x-M_{1}\right)}^{2}\cdot I\left(x\right)\;dx \end{array}$$

The integrals are calculated over the same horizontal direction *x* in Figure 3a. As the beamlet interact with the sample, the induced transmission (*T*), refraction (*R*), and scattering (*S*) can be calculated by the following simple relations:

(2a) $$\begin{array}{cc} T & =M_{0}^{sample}/M_{0} \end{array}$$

(2b) $$\begin{array}{cc} R & =M_{1}^{sample}-M_{1} \end{array}$$

(2c) $$\begin{array}{cc} S & =M_{2}^{sample}-M_{2} \end{array}$$

where the $M_{0}^{sample}$, $M_{1}^{sample}$, and $M_{2}^{sample}$ are the moments calculated with the sample in the field of view. The images and data in Figure 3b,c were obtained using this method.

The gaussian interpolation method is based instead on beamlet interpolation in the following gaussian equations:

(3a) $$\begin{array}{cc} I(x) & =\frac{A}{\sqrt{2\pi \sigma^{2}}}exp\left[-\frac{{\left(x-x_{0}\right)}^{2}}{2\sigma^{2}}\right] \end{array}$$

(3b) $$\begin{array}{cc} I_{sample}\left(x\right) & =\frac{tA}{\sqrt{2\pi (\sigma^{2}+\sigma_{sample}^{2})}}exp\left[-\frac{{\left(x-x_{0}-\Delta x_{ref}\right)}^{2}}{2(\sigma^{2}+\sigma_{sample}^{2})}\right] \end{array}$$

with 3a and 3b interpolating the beamlets without and with sample, respectively. In these equations $\vec{x}$ is the Gaussian's spatial extension vector and *A*, $x_{0}$, and σ are its amplitude, center and width in absence of sample. When a sample is introduced, the curve is attenuated by the factor *t*, shifted by $\Delta x_{ref}$, and its width is changed by $\sigma_{sample}$. By fitting every beamlet to Equations 3a and 3b it is possible to find *t*, $\Delta x_{ref}$, and $\sigma_{sample}$ and to generate three independent images of the samples: attenuation, refraction, and scattering, respectively.

The CT scans were analyzed using the Gaussian fitting method on each individual projection. The projections were then reconstructed into transversal slices by inverse Radon transforms via python‐supported scikit‐image library. The resulting artifacts were blurred via selective blurring of the affected pixels using a Gaussian filter applied over five slices (approximately 25 μm). In addition, residual artifacts were mostly removed by intensity‐based thresholding, as their signal levels were consistently lower than those of relevant features. The slices were then rendered into 3D volumes using commercially available software: Avizo for the mouse lung and OSC Dragonfly for the esophagus. CNRs were evaluated using volumetric ROIs encompassing five adjacent slices, providing a larger statistical sample for the analysis of the background ROIs (dashed white boxes in 4; 75 000 voxels) and the features (red and cyan boxes; 750 and 12 500 voxels, respectively). These features were selected to ensure a direct and reliable comparison between modalities, as they remain clearly identifiable despite the high structural inhomogeneity of the biological sample.

The BT imaging system was simulated in Python using a wave‐based approach based on the Fourier integral method [66], incorporating the previously described source and detector parameters. The mask dimensions used were those obtained from the characterization process. The energy spectrum delivered by the beam was taken from SpekPy 2.0.8 online toolkit inputting the source's characteristics. The mask, wire, and detector scintillator materials were taken from the Xraylib library. The simulation results, shown in Figure 3d, were compared with experimental data.

## Conflicts of Interest

A. Olivo is a named inventor on patents owned by UCL protecting the imaging technology used to obtain the presented results.

## Supporting information


**Supporting File**: advs77561‐sup‐0001‐SuppMat.docx.

## Data Availability

The data that support the findings of this study are available from the corresponding author upon reasonable request.
