# Supplementary material for: Microfluidic Gratings for X‐Ray Phase Contrast Imaging
Source: Adv Sci (Weinh). 2026 Sep 8:e77561. Online ahead of print. doi: 10.1002/advs.77561 (PMC13554524; doi:10.1002/advs.77561)
Supplement: Supplementary file 1 [file ADVS-9999-e77561-s001.docx]

**Supplementary Information for “Microfluidic gratings for X-ray Phase Contrast Imaging”**

Alessandro Rossi^1^ Antonio Ferraro^2^ Francesco Coccimiglio^3^ Tiziana Ritacco^4^ Alberto Astolfo^5^ Michele Giocondo^2,3^

Vincenzo Formoso^2,3^ Raffaele Giuseppe Agostino^2,3^ Francesco Iacoviello^6^ Ioannis Papakonstantinou^1^ Alessandro Olivo^5^*

^1^Photonic Innovations Lab, Department of Electronic & Electrical Engineering, University College London, Torrington Place, London, WC1E 7JE UK

^2^Consiglio Nazionale delle Ricerche - Istituto di Nanotecnologia CNR-NANOTEC, Rende, 87036, Italy

^3^Department of Physics and STAR Research Infrastructure, University of Calabria, Via Tito Flavio, Rende, 87036, CS, Italy

^4^Consiglio Nazionale delle Ricerche - Istituto di Microelettronica e Microsistemi, Rome, 00133, Italy

^5^Department of Medical Physics and Biomedical Engineering, University College London, Gower Street, London, WC1E 6BT UK

^6^University College London, Electrochemical Innovation Lab, Department of Chemical Engineering, London, WC1E 7JE, UK

Slice 500

Slice 1000

**Figure S2:** a) transmission (above) and refraction (below) images of sapphire, boron with a W-core, and nylon wires; b) transmission (left) and refraction (right) profiles of the boron wire; c) transmission (left) and refraction (right) profiles of the nylon wire.

**Figure S1:** attenuation-based reconstruction of two CT-slices showing the microfluidic channels’ cross sections.
